# Spatiotemporal and Long Lasting Modulation of 11 Key Nogo Signaling Genes in Response to Strong Neuroexcitation

**DOI:** 10.3389/fnmol.2017.00094

**Published:** 2017-04-11

**Authors:** Tobias E. Karlsson, Katrin Wellfelt, Lars Olson

**Affiliations:** Department of Neuroscience, Karolinska InstitutetStockholm, Sweden

**Keywords:** NgR1, Nogo-A, Lotus, Lingo-1, Troy, Lgl1, BDNF, kainic acid

## Abstract

Inhibition of nerve growth and plasticity in the CNS is to a large part mediated by Nogo-like signaling, now encompassing a plethora of ligands, receptors, co-receptors and modulators. Here we describe the distribution and levels of mRNA encoding 11 key genes involved in Nogo-like signaling (Nogo-A, Oligodendrocyte-Myelin glycoprotein (OMgp), Nogo receptor 1 (NgR1), NgR2, NgR3, Lingo-1, TNF receptor orphan Y (Troy), Olfactomedin, Lateral olfactory tract usher substance (Lotus) and membrane-type matrix metalloproteinase-3 (MT3-MPP)), as well as BDNF and GAPDH. Expression was analyzed in nine different brain areas before, and at eight time points during the first 3 days after a strong neuroexcitatory stimulation, caused by one kainic acid injection. A temporo-spatial pattern of orderly transcriptional regulations emerges that strengthens the role of Nogo-signaling mechanisms for synaptic plasticity in synchrony with transcriptional increases of BDNF mRNA. For most Nogo-type signaling genes, the largest alterations of mRNA levels occur in the dentate gyrus, with marked alterations also in the CA1 region. Changes occurred somewhat later in several areas of the cerebral cortex. The detailed spatio-temporal pattern of mRNA presence and kainic acid-induced transcriptional response is gene-specific. We reveal that several different gene alterations combine to decrease (and later increase) Nogo-like signaling, as expected to allow structural plasticity responses. Other genes are altered in the opposite direction, suggesting that the system prepares in advance in order to rapidly restore balance. However, the fact that Lingo-1 shows a seemingly opposite, plasticity inhibiting response to kainic acid (strong increase of mRNA in the dentate gyrus), may instead suggest a plasticity-enhancing intracellular function of this presumed NgR1 co-receptor.

## Introduction

**Figure 1:**
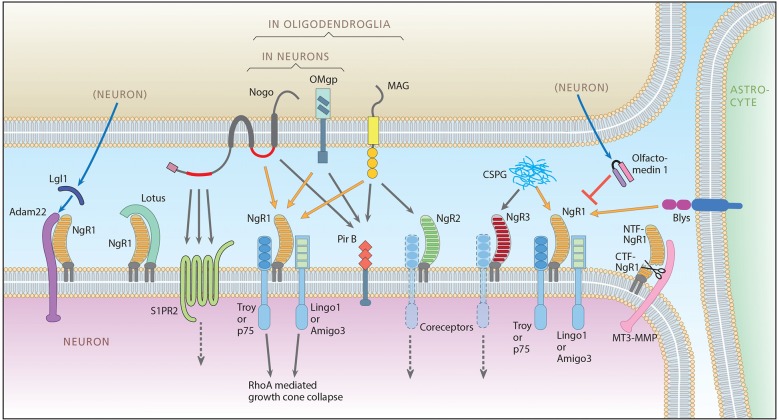
**The Nogo signaling landscape.** The figure illustrates signaling interactions taking place between an upper cell that can be a neuron or an oligodendrocyte, a lower neuron, an astrocyte (right) and the extracellular compartment (Chondritonin Sulfate Proteo glycan, CSPG). For clarity the lower neuron is depicted as the receptor side, but it should be noted that the directionality with regard to pre/post and autocrine is not yet clear. Ligands include Nogo A with two extracellular receptor binding domains, Nogo66 (red right segment) and Nogo-A-Δ20 (red left segment), Oligodendrocyte-Myelin glycoprotein (OMgp), both expressed by neurons and oligodendrocytes, Myelin-Associated Glycoprotein (MAG), expressed only in oligodendroglia, B-lymphocyte stimulator (Blys), expressed in astrocytes and CSPG present in the extracellular matrix (and on cell membranes, not shown). Main ligand-receptor signaling is indicated by orange arrows, additional signaling by gray arrows. Receptors include Nogo receptor 1 (NgR1), NgR2 and NgR3, Sphingosine 1 Phosphate Receptor 2 (S1PR2), and Paired immunoglobulin-like receptor B (PirB). Glycosylphosphatidylinositol (GPI)-anchored Nogo receptors need co-receptors: for NgR1, co-receptors are thought to be TNF receptor orphan Y (Troy) or p75 (also functioning as the low-affinity neurotrophin receptor), and Lingo-1 (LRR and Ig domain-containing, Nogo Receptor-interacting protein) or Amphoterin-induced gene and ORF-3 (Amigo-3). Whether Lingo-1 operates in the cell membrane or in the cytoplasm is however not clear (Meabon et al., 2015). Known modulators of Nogo-like signaling include: a disintegrin and metalloproteinase (Adam 22) that associates with NgR1 and is presumably integrated in the postsynaptic membrane and linked to Post-Synaptic Density 95 (PSD95). NgR1 facilitates the binding of secreted leucine-rich, glioma inactivated protein 1 (LgI1) to Adam22, and the Adam22-LgI1 complex inhibits Nogo A signaling. Lateral olfactory tract usher substance (Lotus), binds to NgR1, thereby inhibiting NgR1-mediated signaling. Lotus does not bind to NgR2 or NgR3. Olfactomedin (also known as pancortin in mice) is a secreted glycoprotein that reduces the binding of NgR1 to co-receptors p75 and Lingo-1, thereby inhibiting NgR1 signaling. Membrane-type matrix metalloproteinase-3 (MT3-MMP) associates with NgR1 and cleaves it into a soluble N-terminal ectodomain fraction consisting of amino acids 1-358 (NTF-NgR1) that can bind to, and thus block Nogo, and a C-termimal fraction (CTF-NgR1) which cannot bind ligands (Designed by L. Olson and T. Karlsson, made by A. Röhl).

The formation of lasting memories is the result of a large number of interacting mechanisms. Changes occur on the epigenetic level (Halder et al., 2016), there is formation of new neurons through neurogenesis (Frisén, 2016; Poo et al., 2016), and there are glial alterations, including white matter changes. However, there is strong evidence that the final executive representation of a novel engram is an altered structural pattern of the synaptic network. Synaptic plasticity in the brain was first demonstrated in response to denervations (Raisman, 1969; Cotman et al., 1973) and later found to occur also in association with long term potentiation (Yuste and Bonhoeffer, 2001). *In vivo* imaging of newly formed postsynaptic sites in rodents suggests that individual synapses can exist for years, as required if the altered synaptic pattern is to carry lasting memories (Yang et al., 2009). Such alterations of synaptic patterns and the preservation of needed synapses are regulated by the concerted action of a large number of gene products. There is a prominent role for nerve growth stimulating factors, in particular BDNF (Lu et al., 2014), and nerve growth inhibiting factors, not least the Nogo signaling system. Pioneered by Schwab and coworkers Nogo-type signaling is now known to encompass a complex set of ligands, receptors, co-receptors and possible agonists and antagonists (Schwab, 2010; Ferraro et al., 2011; Sato et al., 2011; Akbik et al., 2012; Mironova and Giger, 2013) as summarized in Figure 1.

Nogo receptor 1 (NgR1) is downregulated by increased neuronal activity (Josephson et al., 2003), and lack of NgR1 causes ocular dominance plasticity to persist into adulthood (McGee et al., 2005). We hypothesized that NgR1 regulates long term memory formation and showed that overexpression of a NgR1 transgene severely impairs memory formation (Karlén et al., 2009), dendritic architecture and spine densities (Karlsson et al., 2016). This is in agreement with several other groups who have showed altered structural plasticity and memory effects by modulating Nogo signaling (Delekate et al., 2011; Wills et al., 2012; Akbik et al., 2013; Petrasek et al., 2014b; Zemmar et al., 2014; Bhagat et al., 2016; Zagrebelsky et al., 2017).

We subsequently described how increased neuronal activity, as induced by kainic acid, upregulates the transcriptional activity of Nogo receptor 2 (NgR2), NgR3, and the NgR1 antagonist Lotus immediately following a downregulation of NgR1. We hypothesized that this would increase local structural synaptic plasticity in the neuropil, while minimizing the risk of neurite extensions escaping the local environment (Karlsson et al., 2013). Transcription of members in the Nogo family are also regulated by ECT (Nordgren et al., 2013) and spinal cord injury (Endo et al., 2009).

Understanding the roles and regulations of genes involved in Nogo-type signaling should offer multiple opportunities to develop plasticity-promoting treatments. However, detailed knowledge about how the many genes now known to be involved in Nogo-type signaling are regulated by a strong neuroexcitatory stimulus is lacking. Such knowledge is also needed to understand the possible roles of Nogo-type signaling during different phases of memory formation and consolidation. A deeper understanding would also be relevant to pathologies such as post-traumatic stress disorder.

Kainic acid can be used as a tool to induce strong neuronal excitation. Depending on dosing and route of delivery, the drug has been used to excite neurons, as an excitotoxin, as well as to model epilepsy (Jonsson, 1980). Here we chose to use kainic acid as a pharmacological tool to obtain strong, simultaneous, brain-wide neuronal excitation, while avoiding prolonged epileptic periods. Several new genes implicated in Nogo-type signaling have been identified. In this study, we map the location and activity-dependent temporal and regional changes in these genes as reflected by regional mRNA levels, determined by radioactive quantitative *in situ* hybridization. We also present higher temporal resolution of previously studied genes. The findings allow us to provide a longitudinal and regional overview of 11 out of the identified 19 genes involved in Nogo-type signaling (Figure 1). In total, nine different brain areas were examined under normal conditions and during the first 3 days after a strong neuroexcitatory stimulus. We also compare the location and time course of these alterations to those of mRNA encoding BDNF and the housekeeping gene GAPDH. We find that the patterns of transcriptional alterations of genes coding for ligands, receptors, co-receptors and modulators differ between genes and brain areas and over time in a manner that strengthens a role for Nogo-type signaling as a dynamic, strongly activity-regulated, plasticity-implicated system.

## Materials and Methods

### Ethics Statement

The experiments were approved by the Stockholm North animal ethics committee (N580/11).

### Animals

Male mice (C57/Bl6) were purchased from Charles-River (Sulzfeld, Germany) at 12 weeks of age. This strain was chosen as it is the background strain used (or backcrossed to) for most of the genetic manipulations including our NgR1 overexpressing mice. This strain is also the one mostly used in studies of plasticity, regeneration and behavior. Mice were group housed in litters of 5/cage with access to food and water *ad libitum*. All mice were kept in the same animal room on a 12/12 h light/dark cycle (lights on 06:00–18:00) and temperature was kept at 22–23°C with a relative humidity of 60%. Each cage had a small paper house and some paper towels for “enrichment”.

### Kainic Acid Administration

The drug was administrated as a single injection in a dose that causes strong neuronal activation (30 mg/kg i.p.) without resulting in significant neuronal loss (Schauwecker and Steward, 1997). Mice were injected in and kept in their home cage for the rest of the experiment. Following injection, the behavior of the animals was monitored by two experienced scientists and scored based on a standardized 6-grade seizure scale (Schauwecker and Steward, 1997). Only mice graded IV (rearing and falling over) or V (repeatedly rearing and falling over) were included in the subsequent analysis. No difference in the seizure scores was seen between kainic acid groups. The groups receiving the kainic acid injection is referred to as the induced neuronal activity group. They were compared to mice without intervention, to investigate how an episode of strong neuronal activation alters baseline mRNA levels found in untreated controls.

### Tissue Preparation

Mice were sacrificed (by decapitation) at the following time points; 1 h (*n* = 7), 2 h (*n* = 6), 4 h (*n* = 5), 8 h (*n* = 6), 12 h (*n* = 5), 16 h (*n* = 6), 24 h (*n* = 6) and 72 h (*n* = 6) after the kainic acid injection. As a control group, untreated mice (*n* =13), denoted “0” h, were used. Following dissection, brains were immediately frozen on dry ice and kept at −80 °C until sectioning. Brains were serially sectioned (section thickness 14 μm) at levels containing striatum and hippocampus, using a cryostat (Microm).

### Quantitative *In Situ* Hybridization

For *in situ* hybridization, three sections from a striatum level and three from a hippocampus level per mouse were used. For each gene every 20th section was used and all genes were analyzed in the same cohort of animals. We performed *in situ* hybridization as previously described (Dagerlind et al., 1992) using ^33^P-labeled oligonucleotide antisense DNA probes. When designing these probes we first used the UCSC genome browser^1^) to verify that the probe only matched the desired mRNA. After confirming a unique match, we used Mfold (Version 3.2; Mathews et al., 1999; Zuker, 2003) to verify that the probe was not likely to form hairpin loops. For each specific mRNA, several probes were created for different parts of the targeted mRNA. The oligonucleotide sequences used in the present study are available from the authors. We analyzed the hybridization patterns of the candidate oligonucleotide probes before one was chosen for the experiments (expression patterns were always highly similar for probes directed at the same transcript, but some probes generated stronger signals and were chosen for subsequent experiments); expression patterns were also compared to previously published data when available.

**Figure 2:**
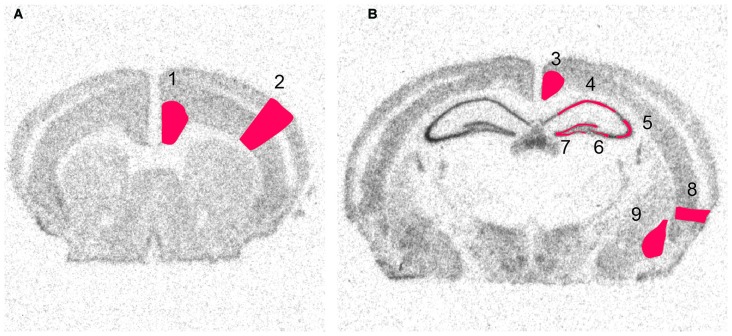
**Scanned images from X-ray film autoradiographs.** Examples from *in situ* hybridization with a ^33^P-labeled oligonucleotide. These high resolution scans were used for densitometry of defined areas. Shown is the distribution of radioactivity obtained by hybridization to GAPDH mRNA at two levels of the brain. Framed areas indicate measured areas of interest at **(A)** a rostral and **(B)** a caudal level. 1, Cingulate cortex, 2, Sensory cortex, 3, Retrosplenial cortex, 4, CA1, 5 Lateral CA3, 6, Medial CA3, 7, Dentate Gyrus, 8, Enthorhinal Cortex, 9, Amygdala.

**Figure 3:**
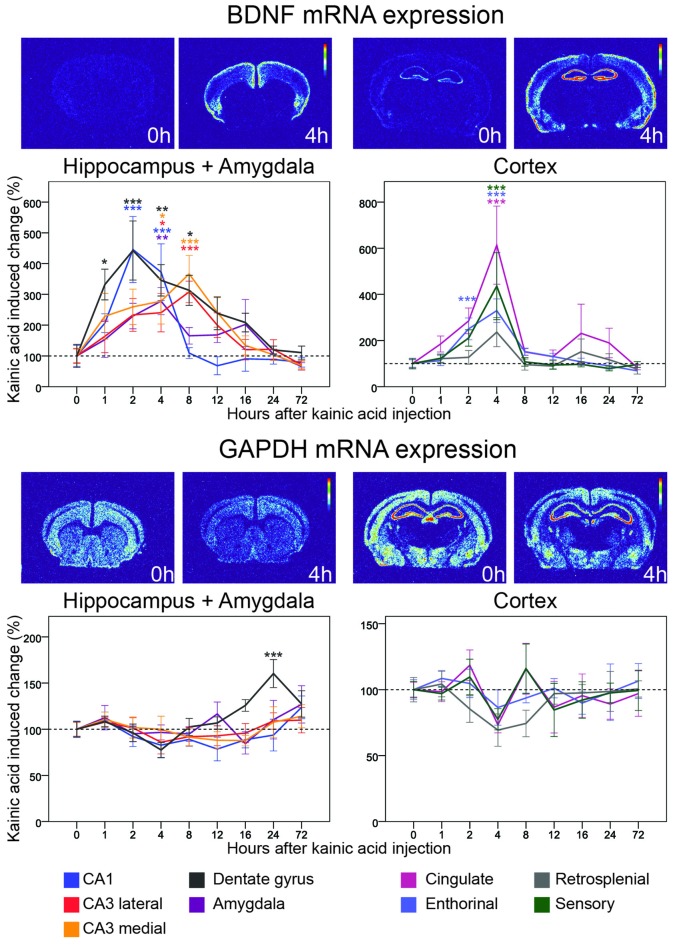
**Three day time course of alterations of BDNF and GAPDH mRNA in response to kainic acid.** In this and the following figures, two pairs of heatmaps, both depicting a control and a kainic acid treated mouse, at a striatal and a dorsal hippocampal level, respectively, are shown för each gene. In each pair the heatmap from the kainic acid treated animal is chosen at a time after treatment when the most visible effects were noted. Time after treatment is indicated. Control brain heatmaps are labeled 0 h. The graphs show alterations of mRNA levels relative to untreated controls in response to kainic acid at eight time points in nine brain areas. Note that to visualize details during the first several hours, the X-axis is non-linear. Mean ± SEM. *p < 0.05, **p < 0.01, ***p < 0.001. Color of asterisks refer to the corresponding brain region.

**Figure 4:**
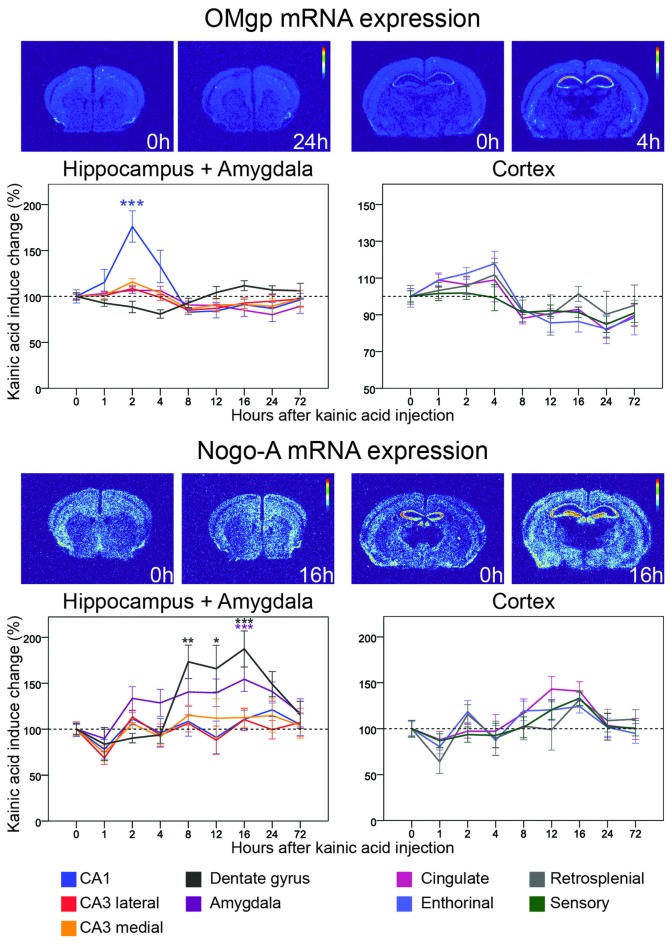
**Three day time course of alterations of OMgp and Nogo-A mRNA in response to kainic acid.** For legend, see Figure 3.

**Figure 5:**
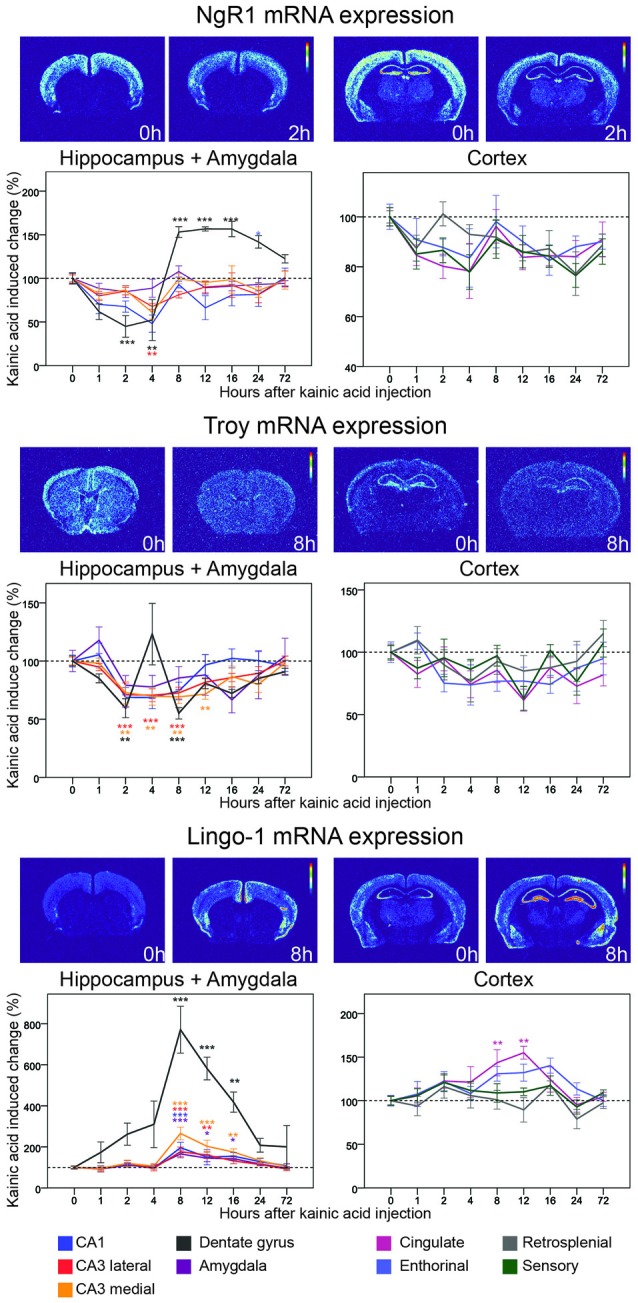
**Three day time course of alterations of NgR1, Troy and Lingo-1 mRNA in response to kainic acid.** For legend, see Figure 3.

**Figure 6:**
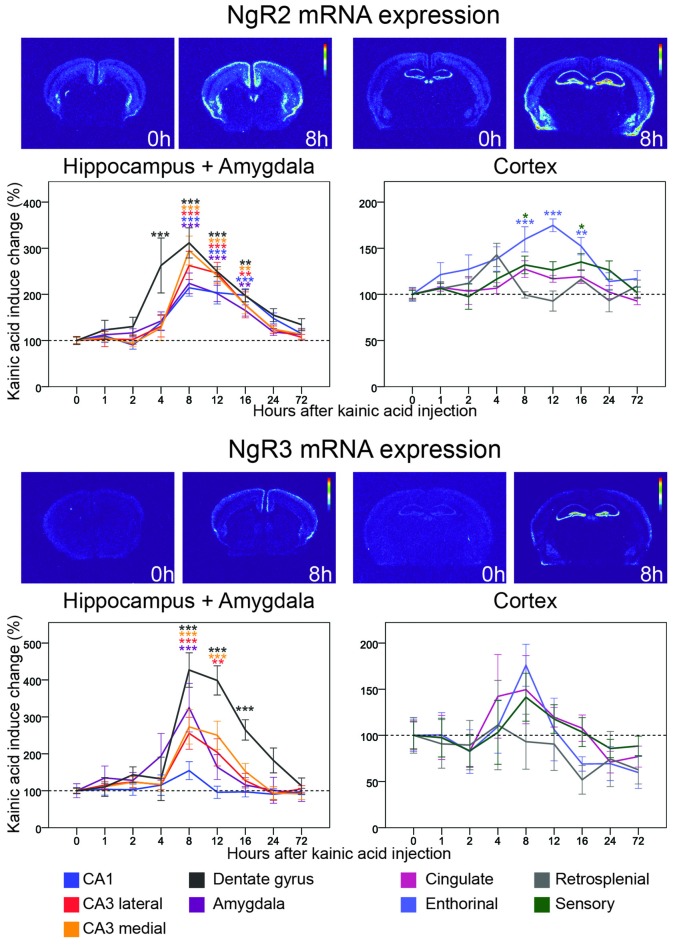
**Three day time course of alterations of NgR2 and NgR3 mRNA in response to kainic acid.** For legend, see Figure 3.

**Figure 7:**
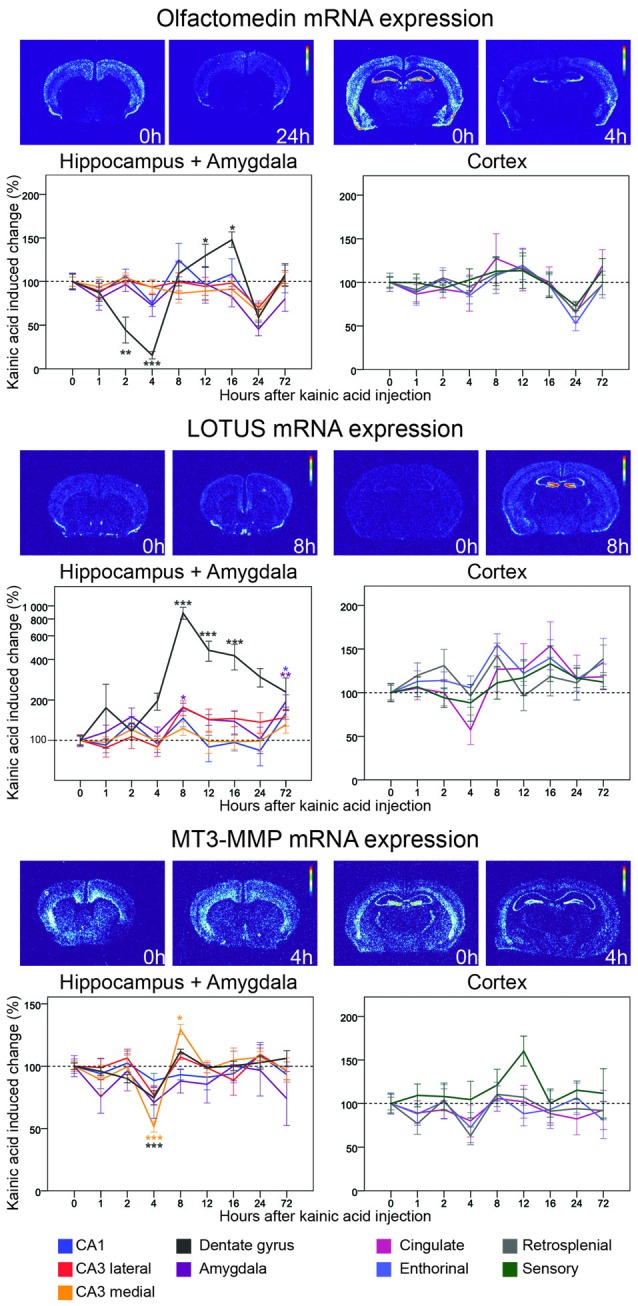
**Three day time course of alterations of Olfactomedin, Lotus and MT3-MMP mRNA in response to kainic acid.** For legend, see Figure 3.

Following *in situ* hybridization, slides were put on X-ray film (Biomax, Eastman Kodak) for 3–28 days (depending on the strength of expression) before being developed. The films were digitized using a high resolution scanner (v750 PRO, Epson) and expression was quantified using an image analysis program (ImageJ v. 1.32j^2^). The scanned files were not modified in any way prior to analysis. In each run a ^14^C standard curve (Amersham) was included and used to convert the measured expression to linearized data. In total nine areas were examined with three sections per mouse. A few sections had to be excluded due to drying of the slides during the hybridization process. In total over 19,000 manual measurements were collected. For *in situ* images shown in Figures 3–7, the raw images were converted to heatmaps using the standard curve in ImageJ (Péan, 2012). The same settings were used for all images of a specific gene. Figure 2 shows examples of the original scanned film results and the measured areas of interest. The nine brain areas were chosen based on their involvement in plasticity related events such as memory and cognition. Hippocampus, the dentate gyrus and amygdala were chosen due to their prominent roles in memory and due to clear expression of the genes of interest. In cortex cerebri, we limited analysis to the entorhinal cortex, and the retrosplenial cortex, due to their involvement in memory, and the cingulate cortex due to its strong role in emotional processing. The sensory cortex was included as a region that should become strongly activated by kainic acid injection, but presumably be less plastic. All original images are available from the authors.

### Statistics

Data were analyzed using a mixed linear model with Bonferroni correction for the number of regions analyzed (9). When a significance between group effects was found, this was followed up with pairwise testing of all groups to the 0 h group using the estimated marginal means and Bonferroni correction using SPSS. In the text, the significance presented will refer to the between group effects if nothing else is stated. For visualization, all data were normalized to the 0 h group.

## Results

**Figure 8:**
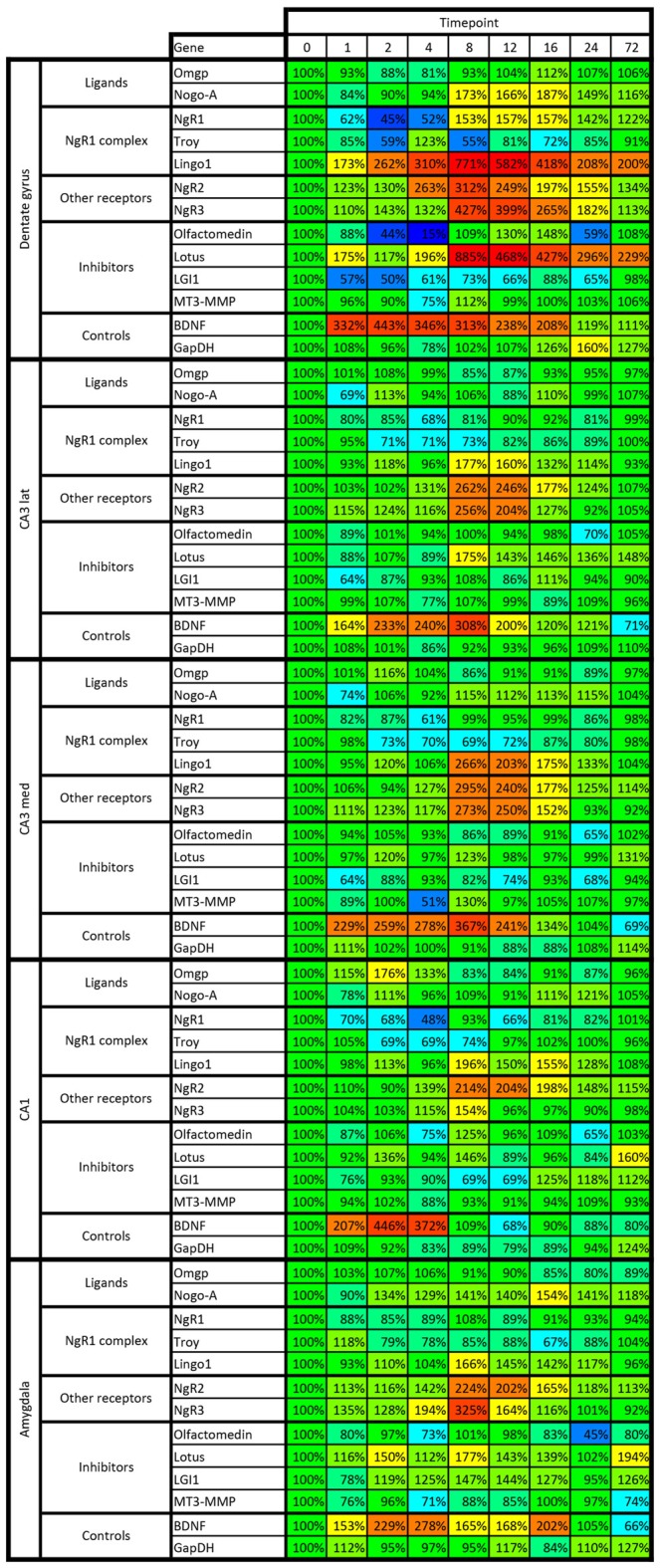
**Heat map of kainic acid-induced changes of mRNA levels as transcribed from all 13 investigated genes in the dentate gyrus, CA1, medial and lateral CA3 and amygdala.** Changes are expressed as percent of control levels 1–72 h after kainic acid. Colors indicate degree of increase (warmer colors) or decrease (colder colors). Note e.g., that the dentate is more affected by kainic acid than other areas, both in terms of increased and decreased levels of mRNA species.

**Figure 9:**
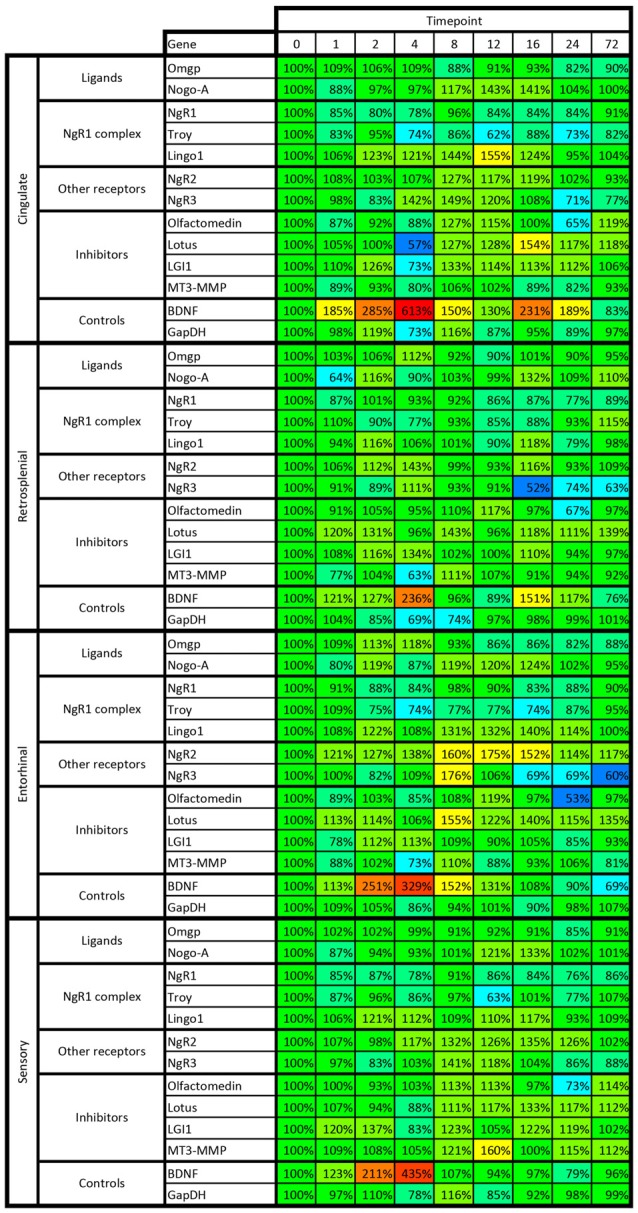
**Heat map of kainic acid-induced changes of mRNA levels as transcribed from all 13 investigated genes in cingulate, retrosplenial, entorhinal and sensory cortex.** Changes are expressed as percent of control levels 1–72 h after kainic acid. Colors indicate degree of increase (warmer colors) or decrease (colder colors). Note e.g., that BDNF mRNA is strongly increased in all cortical areas 2–4 h after kainic acid, while NgR1 is slightly below 100% in 31 of the 32 data points. For color code, see Figure 8.

Mice were injected with kainic acid in their home cage and kept for up to 72 h before being sacrificed and processed for *in situ* hybridization. There was no difference in the seizure scores between groups injected with kainic acid (data not shown). We analyzed the expression of all genes in nine brain areas connected to emotional processing and memory. For each gene, the results were normalized to mice without any treatment, referred to as the “0 h” time point, and all data are represented as percentage change from 0 h. Thus, we are comparing how strong neuronal activation by the kainic acid treatment changes activity of the chosen genes compared to mice without any intervention. In the following, we will describe alterations of individual genes. All results are also summarized in Figures 8, 9.

### Activity and Expression Controls: BDNF and GAPDH

#### BDNF mRNA

The neurotrophin BDNF is expressed mainly in cortical and hippocampal brain areas (Ernfors et al., 1990) and of key importance for brain plasticity (Lu et al., 2014). BDNF mRNA levels are typically relatively low in the non-perturbed brain. Validating the current study protocol, we found fast and marked kainic acid induced increase of BDNF mRNA levels in almost all investigated areas (Figure 3). A closer look at the time course revealed that the rise of BDNF mRNA was faster in parts of the hippocampal formation than in cortical areas with clear increases 1 h after the KA challenge and peak levels after 2 h in the dentate gyrus and CA1 region. BDNF mRNA levels in medial and lateral CA3 regions peaked at 8 h. CA1 BDNF mRNA levels had returned to baseline by 8 h; by 24 h, BDNF mRNA levels in the dentate gyrus, and in the medial and lateral CA3 had also returned to baseline levels.

Marked increases of BDNF mRNA were also found in several cortical areas. Interestingly, these changes all peaked at 4 h, hence 2 h later than in the dentate gyrus and CA1. The largest increase was seen in cingulate (>6-fold) and S1 sensory (>4-fold) cortex, lesser increases were noted in enthorhinal and retrosplenial cortex. The duration of effects in cortical areas and CA1 was 8 h, as opposed to 12–16 h in CA3 and the dentate gyrus. BDNF mRNA levels were also increased in amygdala during the first 16 h with a peak at 4 h.

#### GAPDH mRNA

We used GAPDH mRNA hybridization to serve as an expression control in all areas of interest. In the non-stimulated brain, GAPDH mRNA was robustly found in cortical and hippocampal formation neurons. The levels of GAPDH mRNA did not change significantly at any time in any area except for a late, significant 50% increase (*p* < 0.001) in the dentate gyrus peaking at 24 h after the kainic acid challenge (Figure 3). This indicates that overall transcription was not increased due to treatment.

### Neurally Expressed Inhibitory Ligands: OMgp and Nogo-A

#### OMgp mRNA

Oligodendrocyte-Myelin glycoprotein (OMgp) is expressed by both neurons and oligodendroglia and serves as a ligand for NgR1 and PirB (Wang et al., 2002; Atwal et al., 2008). Relatively low OMgp mRNA hybridization signals were noted in most cortical areas, although a stronger signal was noted in piriform cortex. OMgp mRNA levels were not significantly altered in either the dentate gyrus or CA3. However, levels were upregulated in CA1 (76%, *p* < 0.001) 2 h after kainic acid administration, after which levels dropped again, reaching baseline levels by 8 h (Figure 4). The situation was different in amygdala where mRNA levels remained stable during the initial part but dropped slightly by 24 h (20% down-regulation, *p* = 0.015). Likewise, OMgp mRNA levels were significantly downregulated in perirhinal and cingulate cortex in the later phase of the experiment (Figure 4). An overview of all nine investigated areas shows that the early increase of OMgp mRNA in CA1 stands out as the most marked effect of kainic acid.

#### Nogo-A mRNA

This ligand, originally identified in myelin (Caroni and Schwab, 1988), is also expressed by neurons (Josephson et al., 2001). Unlike OMgp mRNA, Nogo-A mRNA was widely expressed in the untreated brain and present at higher levels than OMgp mRNA. Nogo-A mRNA levels were rather stable over time and across brain areas. However, levels were significantly, albeit modestly affected in the dentate gyrus and amygdala (Figure 4). In the dentate gyrus Nogo-A mRNA was significantly upregulated between 8 h and 16 h after kainic acid (peak 16 h 88% increase, *p* < 0.001). In amygdala Nogo-A increased slowly, reaching a peak upregulation at 16 h (56% increase, *p* = 0.007) and then returned to baseline levels by 72 h. Observing all nine investigated areas, a pattern emerges with a general modest decrease at 1 h. This is followed by a modest steadily increase until 16 h after kainic acid, after which there is a decline of Nogo-A mRNA in all areas towards pretreatment levels by 72 h.

### The NgR1 Signaling Complex: NgR1, Lingo and Troy

#### NgR1 mRNA

NgR1 was the first identified Nogo receptor (Fournier et al., 2001), and binds the Nogo66 segment of Nogo, as well as OMgp (Wang et al., 2002) and MAG (Liu et al., 2002). Its expression is exclusively neuronal (Fournier et al., 2001; Josephson et al., 2002). As expected, we find robust, gray matter-specific expression of NgR1mRNA in cerebral cortex, amygdala, habenula and the hippocampal formation of untreated animals. Lesser amounts of NgR1 mRNA were found in thalamus, while striatum did not express detectable amounts of NgR1 mRNA. NgR1 mRNA levels were found to be significantly altered in two regions, the hippocampal CA1 area, and the dentate gyrus (Figure 5). In CA1, levels had declined already at the first studied time point, 1 h, and continued to drop until 4 h when levels were down by 52% (*p* = 0.002). Expression of NgR1 mRNA in CA1 had returned to baseline levels at the 8 h timepoint, and no further significant changes were seen in CA1. In the dentate gyrus, NgR1 mRNA levels also decreased during the early phase and thus mirrored changes in CA1, but with a slightly faster time course and larger amplitude. Expression was significantly downregulated at 1 h (*p* = 0.014) and a maximal down-regulation was seen after 2 h when there was a 55% reduction of mRNA levels (*p* < 0.001) lasting until 4 h. In contrast to what was found in CA1, the NgR1 mRNA signal then increased markedly in the dentate gyrus, overshooting the baseline level by 53% at 8 h (*p* < 0.001), whereafter the level remained upregulated at least until the 24 h timepoint. Hence the duration of the decrease of NgR1 mRNA in the dentate gyrus was 4 h, while the following increase lasted at least 24 h and possibly much longer.

By and large, all four analyzed cortical areas, cingulate, enthorinal, retrosplenial and sensory cortex, undergo a modest but not significant decrease of NgR1 mRNA. This is similar to that noted for CA1, CA3 and amygdala, although NgR1 mRNA levels in cortical areas appear not to have returned to pre-kainic acid levels at 72 h (31 of 32 measured time points in cortical areas are <100%, Figure 9). When changes of NgR1 mRNA levels of all investigated areas are summarized, the dentate gyrus stands out with a rapid decrease of Ngr1 mRNA levels, followed by a marked, lasting increase well above baseline, a pattern not seen in any other investigated brain area (Figure 5).

#### Lingo-1

Being GPI-linked to the cell membrane, the NgRs depend on co-receptors for signaling. Lingo-1 has been identified as a NgR1 co-receptor, interacting with another co-receptor, p75 and NgR1 (Mi et al., 2004). However, more recent work questions the site of action of Lingo-1, suggesting an intracellular role instead (Meabon et al., 2015). Untreated mice had robust presence of mRNA encoding the presumed NgR1 co-receptor Lingo-1 in cerebral cortex (Figure 5) and in CA1 and CA3, while the granule cell layers in the dentate gyrus had a lower expression of Lingo-1 mRNA. The strongest hippocampal expression was seen in CA3 (Figure 5). Strikingly, Lingo-1 mRNA expression levels were significantly increased in six out of nine regions by the kainic acid challenge. The most profound relative change was seen in the dentate gyrus were mRNA was strongly upregulated at all time points after KA stimulation, peaking at 8 h (771% upregulation, *p* < 0.001). It should be noticed, however, that this almost eight-fold increase can be partly explained by the very low pre-kainic acid levels of Lingo-1 mRNA in the dentate gyrus. The same pattern of upregulation was seen in investigated hippocampal CA areas, all three of which showed a significant upregulation 8 h after stimulation. The magnitude and time course of upregulation was greatest in medial CA3 (165% at 8 h, 74% at 16 h) and lowest in CA1 (96% at 8 h, 54% at 16 h). An upregulation of Lingo-1 was also seen in amygdala (*p* = 0.001) with a time course and magnitude similar to that seen in hippocampus. Lingo-1 was also significantly upregulated in cingulate cortex but the time course was a bit slower than in previously mentioned areas, with a maximum reached at 12 h (54%, *p* = 0.001).

#### Troy

We next assessed TNF receptor orphan Y (Troy), a second co-receptor for NgR1 (Park et al., 2005) that can replace p75. In untreated mice, Troy mRNA was present in the hippocampal formation, cerebral cortex and in many subcortical brain areas. We found Troy mRNA levels to be significantly altered in three regions (Figure 5). Like NgR1, Troy mRNA was rather rapidly downregulated in the dentate gyrus by kainic acid, significantly so at 2 h (41% downregulation, *p* = 0.02). Surprisingly, Troy mRNA levels in the dentate gyrus demonstrated a bi-phasic down/up regulation pattern, returning to baseline or above by 4 h, before being down-regulated again at 8 h (45%, *p* < 0.001). In hippocampus Troy mRNA levels were significantly decreased in all CA subregions with around 30% between 2 h and 8 h, and with the changes in medial CA3 lasting slightly longer and remaining significant at the 12 h timepoint. There were no significant changes in the individual cortical areas examined, or in amygdala. However, an overall view of the nine analyzed areas suggest general modest downregulation of Troy mRNA, and no robust indication of upregulation above baseline levels in any investigated brain area.

### Homologous NgR1 Receptors: NgR2 and NgR3

#### NgR2

Less is known about ligands and co-receptors for the NgR1 related receptors NgR2 and NgR3. NgR2, however, binds MAG, and with higher affinity than NgR1 (Robak et al., 2009). In untreated mice, mRNA encoding NgR2 is present in lower amounts and fewer areas than NgR1 mRNA. There is a scattered presence of NgR2 mRNA in the inner half of the cerebral cortex, and a stronger presence in claustrum and habenula. In the hippocampal formation, CA1 and CA3, but not CA2, express NgR2 mRNA, as does the dentate gyrus. The effects of a kainic acid challenge on NgR2 mRNA levels differed dramatically from those on NgR1 by being upregulated in seven out of the nine examined brain areas. In the dentate gyrus, the mRNA level peaked after 8 h (212% upregulation, *p* < 0.001) and was significantly elevated from 4 h to 16 h after kainic acid. NgR2 mRNA levels in hippocampal areas showed a slightly delayed increase response, after which levels slowly returned to baseline, similar to levels in the dentate gyrus. The first significant upregulation was seen 8 h after kainic acid delivery, and was strongest in medial CA3 (195%, *p* < 0.001) and weakest in CA1 (114%, *p* < 0.001). NgR2 was also significantly upregulated in entorhinal cortex with a peak at 12 h (75%, *p* < 0.001). This is slightly later than the changes seen in the hippocampal formation, and in the somatosensory cortex (32% at 8 h, *p* = 0.028). The hippocampal and cortical upregulations were mirrored in amygdala with a peak at 8 h (124%, *p* < 0.001) and lasting until 16 h after the kainic acid injection.

#### NgR3

NgR3 has been reported to function as a CSPG receptor (Dickendesher et al., 2012). In the untreated mouse, we found low levels of NgR3 mRNA. Similarly to NgR2, NgR3 mRNA levels were strongly upregulated in several brain areas following the strong kainic acid induced increase of neuronal activity. Similar to NgR2, NgR3 mRNA levels were most strongly upregulated in the dentate gyrus, also peaked at 8 h (327%, *p* < 0.001), and remained significantly upregulated until 16 h. Upregulation in medial (173%, *p* < 0.001) and lateral (156%, *p* < 0.001) CA3, was also marked, but with a shorter duration such that expression levels were only upregulated significantly until the 12 h time point. NgR3 mRNA levels in CA1 remained stable, with no significant alteration caused by kainic acid.

NgR3 was also significantly upregulated in amygdala 8 h after kainic acid stimulation (225%, *p* < 0.001). Thus NgR3 mRNA levels were increased the longest in the dentate gyrus, for a shorter time in CA3 and shorter still in amygdala. NgR3 mRNA levels were not significantly altered in the individual cortical areas assessed. Nevertheless, an overall view of all nine investigated brain areas shows that NgR3 mRNA in eight of them peaks 8 h after kainic acid; only retrosplenial cortex lacks such a peak.

### Inhibitors of Nogo-Signaling: LgI1, Lotus, Olfactomedin, MT3-MMP

#### LgI1

When Adam22 is associated with NgR1, this complex attracts the secreted Adam22 ligand LGI1, leading to blockade of NgR1 signaling (Thomas et al., 2010; Figure 1). In the untreated mouse brain, levels of mRNA encoding LGI1 were prominent in CA3 compartments and the dentate gyrus, with robust levels also found in the cerebral cortex and amygdala. However, unlike the situation for all other investigated genes, LGI1 mRNA levels seemed not to be significantly regulated by kainic acid in either high- or low-expressing areas (data not shown).

#### Lotus

By binding to NgR1, Lotus inhibits Nogo-mediated signaling (Sato et al., 2011). In the untreated mouse brain, Lotus mRNA is expressed in many areas, including cortex, hippocampus, the lateral olfactory tract and piriform cortex, but also in striatum, islands of Calleja, and in many subcortical and brain stem nuclei. Levels of mRNA encoding Lotus were dramatically increased in the dentate gyrus with a peak at 8 h (785%, *p* < 0.001) and Lotus mRNA remained significantly elevated for a prolonged period of time. In CA1, Lotus remained close to baseline until it increased significantly at 72 h (60%, *p* = 0.012). In amygdala Lotus had a bi-phasic expression with a peak at 8 h (77%, *p* = 0.012) and at 72 h (92%, *p* = 0.001). Comparing all investigated areas, the strong and lasting increase focally in the dentate gyrus stands out, such that the brain pattern of Lotus activation differs from that of all other genes investigated.

#### Olfactomedin

Originally isolated from frog olfactory neuroepithelium (Snyder et al., 1991), olfactomedin mRNA was later found to be present in rat and mouse brains, including hippocampus and the cerebral cortex and given the alternative name pancortin. This highly conserved, secreted glycoprotein inhibits signaling by the NgR1 complex (Nakaya et al., 2012). We found olfactomedin mRNA to be robustly present in many brain areas, including the cortices, the hippocampal formation and amygdala. Olfactomedin mRNA is also present in striatum and thalamus. Interestingly, this gene seems to have much lower expression in the CA2 area than in other parts of the hippocampus. Following kainic acid treatment, the Olfactomedin mRNA level in the dentate gyrus stands out by undergoing a marked, significant decrease, reaching its lowest level at 4 h, followed by a significant increase to above baseline levels, peaking at 16 h. This pattern is very similar to the changes of NgR1 mRNA levels. Strikingly, Olfactomedin 1 mRNA levels in all nine investigated brain areas are decreased below baseline at 24 h.

#### MT3-MMP

This metalloproteinase associates with NgR1 and cleaves it. The N-terminal fragment is soluble and can bind to Nogo-A, thus blocking it from binding to membrane-bound NgR1 (Ferraro et al., 2011). Messenger RNA encoding MT3-MMP was found in cortical areas, amygdala, hippocampus, striatum and the reticular thalamic nucleus of untreated mice. A striking feature of the MT3-MMP mRNA distribution was that the highest levels were found in the granule cell layer of the dentate gyrus. Kainic acid caused MM3-MMP mRNA to be decrease in medial CA3 at 4 h and then increase to a peak level at 8 h. The same pattern, although smaller in magnitude, was found in the dentate gyrus. MT3-MMP mRNA levels in the lateral CA3 showed a similar, even lesser pronounced, and thus non-significant pattern of alterations. In fact, MT3-MMP mRNA levels were the lowest at 4 h in eight of the nine investigated areas. In cortical areas MT3-MMP mRNA levels were largely stable as were levels in amygdala.

### Additional Genes of Interest

Above we have provided detailed information about location of transcripts and the effects of a strong excitatory stimulus on the transcriptional activity of 11 selected key genes involved in Nogo-like nerve growth inhibitory signaling, as well as BDNF and GAPDH mRNA. We did not observe measurable levels of mRNA encoding S1PR2, PirB, BlyS or p75 in the chosen areas of interest. This remained true even after kainic acid treatment. MAG was not examined due to its very limited expression in the gray matter. Further, we did not investigate the CSPG group of proteins.

## Discussion

The importance of the inhibitory, plasticity-regulating Nogo-like signaling system in gray matter during development and in adulthood has been revealed by studying the effects of perturbations such as blocking, decreasing, or increasing the Nogo-NgR signaling pathway, causing increased and decreased plasticity, respectively (McGee et al., 2005; Park et al., 2006a,b; Lee et al., 2008; Karlén et al., 2009; Wills et al., 2012; Akbik et al., 2013; Tews et al., 2013; Petrasek et al., 2014a; Iobbi et al., 2016; Karlsson et al., 2016; Kellner et al., 2016; Stephany et al., 2016a,b; Zagrebelsky et al., 2017). Increased levels of Nogo-A have been reported in schizophrenia (Novak et al., 2002), multiple sclerosis (Satoh et al., 2005), temporal lobe epilepsy (Bandtlow et al., 2004) and Alzheimer’s disease (Gil et al., 2006). An increasing number of mutations and genetic variants in genes depicted in Figure 1 have been found coupled to neuropsychiatric and neurodegenerative disorders, including schizophrenia (NgR1/Nogo-A: Budel et al., 2008; Willi and Schwab, 2013; Andrews and Fernandez-Enright, 2015), (LGI1/NgR1: Thomas et al., 2016), ALS (NgR1: Amy et al., 2015), Pelizaeus-Merzbacher disease (MAG: Lossos et al., 2015), Parkinson’s disease (Lingo-1: Chen et al., 2015), Tourette syndrome/OCD/ADHD (Olfactomedin: Bertelsen et al., 2015) and epilepsy (LgI1: Fukata et al., 2010); (ADAM22: Muona et al., 2016), further emphasizing the importance of normal Nogo-type control of synaptic plasticity. Current knowledge has also led to the initiations of clinical trials in spinal cord injury (NCT00406016), multiple sclerosis (Ineichen et al., 2017), and ALS in which disease a large phase II trial shows no positive effects of Nogo-A antibodies (Meininger et al., 2014).

To better understand temporal and spatial regulation of Nogo signaling genes, we chose to use a single, strong stimulant, and to compare global changes of multiple genes in several key brain areas known to exhibit plastic responses under physiological conditions. Using this approach, we analyzed the location and activity-induced regulation of levels of mRNA encoding genes identified as important for Nogo type signaling in unprecedented detail. Levels of mRNA representing 11 genes have been quantified in nine different brain regions each, before and at eight time points during the first 3 days after a strong induction of neuronal activity, as compared to mRNA levels in untreated mice. Kainic acid is a strong neuronal stimulant, and toxic if used in too high doses or in particularly sensitive mouse strains. It may also affect non-neuronal cells, such as microglia. Due to its neuroexcitatory efficacy and as based on a wealth of published research, we chose kainic acid to cause a strong pan-cerebral increase of activity in a large number of neurons. It should be noted that kainic acid will also induce activity by secondary means such as release of Ca^2+^ which further increases overall neuronal activity. Hence, it should not be concluded that the effects seen on transcription are due only to direct downstream signaling from kainate receptors, but instead is a summary of the effects of the induced neuronal activation. The stable expression of GAPDH indicates that the treatment was not strongly toxic. Strong upregulation of mRNA encoding BDNF, a key neurotrophin known to be upregulated by increased neuronal activity (Zafra et al., 1992; Josephson et al., 2003), and stable levels of the “housekeeping” gene GAPDH mRNA showed that activity induced genes were upregulated while basal translation was rather unaffected. We find striking gene- and brain area specific differences with respect to alterations of mRNA encoding Nogo-signaling genes, reflecting different effects of the strong neuronal stimulation at the transcriptional level. We compare the effects of the kainic acid injection to untreated controls. Using this setup we cannot determine to which extent the upregulation is due to kainic acid, the saline or the injection itself. Hence, the changes seen compared to untreated mice should be interpreted as due to the combined effects of the injection itself and the kainic acid. Furthermore, while the experiments were planned such that all animals were taken between 08:00 and 20:00, we did not have controls spanning all time points. Hence possible gene expression alterations due to circadian rhythm variations cannot be excluded. However, the fact that most changes were seen either in the rather narrow time-span of 1–4 h, or as slower changes in the window of 8–24 h, suggests that circadian effects, if present, are small, compared to effects of treatment.

Strong and rapid down-regulation of NgR1 mRNA levels occurs in the dentate gyrus in response to neuronal activation (Josephson et al., 2003; Karlén et al., 2009; Wills et al., 2012; Karlsson et al., 2013). However, increased power and better time resolution has now allowed us to discover that NgR1 mRNA levels in the dentate gyrus are not only rapidly decreased in response to neural activation, but thereafter markedly increased well above baseline during a longer phase after kainic acid, possibly working as a homeostatic regulator. This days-long upregulation of NgR1 mRNA levels after the kainic acid challenge was unique to the dentate gyrus. Overall, NgR1 mRNA levels in all other areas were slightly (but not significantly for any individual area) decreased after kainic acid. This possibly suggests a days-long shift towards a temporary status of increased plasticity of hippocampal CA regions, cerebral cortex and the amygdala. We found mRNA encoding the proposed NgR1 co-receptor Troy to be widely expressed in neurons similar to those expressing NgR1 mRNA. Troy mRNA showed a similar but weaker response to neuronal activity in the dentate gyrus as compared to NgR1. The decrease of Troy mRNA also appeared to be more widespread affecting the entire hippocampal formation, and with a tendency of decrease in cortical areas similar to that seen for NgR1 mRNA. General decreases of Troy mRNA are thus in line with decreases of NgR1 mRNA, and we hypothesize that this could allow increased synaptic plasticity.

Lingo-1, the second proposed co-receptor of NgR1, was strongly and widely upregulated following kainic acid stimulation in agreement with earlier findings (Trifunovski et al., 2004). This upregulation is interesting in the light of downregulation of both Troy and NgR1. A recent study has suggested that Lingo-1 is in fact an intracellular protein, which can bind NgR1 (Meabon et al., 2015). Increased intracellular levels of Lingo-1 may internalize NgR1 and/or inhibit intracellular NgR1 from inserting into the cell membrane. This could then contribute to decreased NgR1 mediated signaling. In combination with decreases in transcription of NgR1 itself, and of Troy, this could result in effective temporary silencing of Nogo-like signaling. Again, the magnitude of the kainic acid-induced change is largest in the dentate gyrus, adding evidence to a particularly strong role of Nogo-type regulation of activity-induced plasticity in this region.

The ligands OMgp and Nogo-A show patterns of mRNA alterations that differ from those of NgR1 and its co-receptors. OMgp mRNA levels remain rather stable during the first part of the experiment, except for CA1 where a rapid spike of OMgp mRNA expression was seen. Unlike OMgp, Nogo-A mRNA levels start to rise early in amygdala and continue to be elevated during almost the entire experiment. A few hours later Nogo-A mRNA becomes strongly upregulated in the dentate gyrus. This raises the possibility that OMgp and Nogo-A mRNA increases serve to limit plasticity in CA1 and the dentate gyrus and amygdala, respectively, immediately after an episode of strong neuronal activation. It is interesting that the two areas with the most prominent down-regulation of NgR1 mRNA levels are the same regions where we detect an upregulation of OMgp and Nogo-A mRNA levels. A possible explanation is that early upregulation of the ligands does not in itself activate NgR1 signaling because NgR1 is downregulated. Instead, it could be important for limiting later plasticity when transcription of NgR1 is upregulated. Using our method we cannot determine if it is the same neurons that upregulate OMgp and Nogo-A that also downregulate NgR1.

When it comes to NgR1 inhibitors, levels of mRNA encoding Lotus are massively increased by activity in the dentate gyrus. Hence, NgR1 could be both transcriptionally repressed and actively inhibited by its antagonists. It is interesting that an increase in NgR1 mRNA in the dentate gyrus can be seen at the same time as Lotus mRNA goes up in this area. Together with the regulation of the ligands discussed earlier, it raises interesting questions about how this regulation occurs on a per cell basis and even at subcellular levels. Lotus could: (1) counteract the effects of the late NgR1 upregulation; (2) affect a different cell population than the one that upregulates NgR1 and Nogo-A; or (3) be active in different neuronal compartments.

Unexpectedly, the regulatory time profile of Olfactomedin mRNA levels in the dentate gyrus is similar to that of NgR1 mRNA. However, the phase of upregulation is not as long-lasting as that of NgR1 mRNA. While smaller, the changes in MT3-MMP mirror the changes in Olfactomedin mRNA levels. It may at first seem counterproductive that NgR1 and antagonists downregulate together. Our hypothesis is that during the early stages following activation, NgR1 levels drop strongly in key cells for the memory trace at hand. However, remaining neurons that were not (or only weakly) activated retain higher levels of NgR1 signaling during the early phase. With decreasing levels of the inhibitor Olfactomedin and the reduced proteolytic cleavage by MT3-MMP, inhibition should increase in neurons with high NgR1 levels. Neurons that have strongly downregulated NgR1 should be less affected.

The secreted protein LgI1 interacts with Adam22, a membrane protein that anchors to PSD95 (Fukata et al., 2010; Lovero et al., 2015), and this complex can inhibit NgR1 signaling (Thomas et al., 2010). Mutations in LgI1 that reduce the levels of secreted LgI1 (Senechal et al., 2005), cause “hereditary autosomal dominant partial epilepsy with auditory features” (ADPEAF; Fukata et al., 2010). Animal studies have shown that lack of LgI1 causes lethal epilepsy and that LgI1 regulates excitability in the brain (Fukata et al., 2010). Indeed, mutations of Adam22 has also been linked to severe epilepsy with cerebral atrophy (Muona et al., 2016).

We find that LGI1 mRNA levels are high in the dentate gyrus and CA3, and that lower levels of LgI1 mRNA are present in other hippocampal and cortical areas and amygdala. Unexpectedly, we did not find any significant alteration of the expression of LGI1 mRNA after kainic acid administration in any of the investigated areas (data not shown). The lack of, or minor alterations of LgI1 mRNA levels, shows that LgI1 differs from all other examined genes and suggests that LGI1 inhibition is not significantly regulated by strong neuronal activation. If LgI1 is not significantly regulated, Adam22, or its binding to PSD95 might instead be regulating the Adam22-LgI1 complex. However, our preliminary data have not suggested an activity-induced regulation of ADAM22 mRNA. Together, these findings suggest that the ADAM22-LgI1 complex, important as it may be for regulation of neuronal excitability, is not markedly regulated by alterations of neuronal activity.

The findings with regard to NgR2 and NgR3 mRNA levels extend our previous findings (Karlsson et al., 2013) and show that these two NgR1 receptor analogs are strongly activated by increased neuronal activity in most brain areas. The predominant binding partners to these receptors are MAG (Venkatesh et al., 2005) and CSPGs (Dickendesher et al., 2012) respectively, suggesting a role of restricting plasticity to local areas, by inhibiting plasticity in the presence of myelin or extracellular matrix scaffolds.

We conclude that 10 of the 11 investigated genes involved in different ways in Nogo-type signaling respond to neuronal activation by alterations of mRNA levels during the following 72 h. Each gene is characterized by a unique temporo-spatial pattern of mRNA alterations. Together, patterns of changes and the simultaneous alterations of BDNF mRNA levels can be interpreted as a logical way to temporarily allow optimal plasticity in certain brain regions and neurons, while not affecting other neurons and to also effectively lock altered circuitry of affected neurons in its new conformation, carrying a new engram.

## Author Contributions

TEK, KW and LO designed the experiment. TEK and KW performed the experiments. TEK analyzed the data and performed the statistical tests. TEK, KW and LO interpreted the data and wrote the manuscript. All authors gave final approval of the manuscript.

## Conflict of Interest Statement

The authors declare that the research was conducted in the absence of any commercial or financial relationships that could be construed as a potential conflict of interest.

## Footnotes

^1^http://genome.ucsc.edu/

^2^http://rsb.info.nih.gov/ij/

## References

[B1] Akbik F. Bhagat S. Patel P. Cafferty W. Strittmatter S. (2013). Anatomical plasticity of adult brain is titrated by Nogo Receptor 1. Neuron 77, 859–866. 10.1016/j.neuron.2012.12.027 23473316 PMC3594793

[B2] Akbik F. Cafferty W. B. Strittmatter S. M. (2012). Myelin associated inhibitors: a link between injury-induced and experience-dependent plasticity. Exp. Neurol. 235, 43–52. 10.1016/j.expneurol.2011.06.006 21699896 PMC3189418

[B3] Amy M. Staehlin O. René F. Blasco H. Marouillat S. Daoud H.. (2015). A common functional allele of the Nogo receptor gene, reticulon 4 receptor (RTN4R), is associated with sporadic amyotrophic lateral sclerosis in a French population. Amyotroph. Lateral Scler. Frontotemporal. Degener. 16, 490–496. 10.3109/21678421.2015.1051988 26083872

[B4] Andrews J. L. Fernandez-Enright F. (2015). Genetic variants in Nogo receptor signaling pathways may be associated with early life adversity in schizophrenia susceptibility. BBA Clin. 3, 36–43. 10.1016/j.bbacli.2014.11.008 26673096 PMC4661513

[B5] Atwal J. K. Pinkston-Gosse J. Syken J. Stawicki S. Wu Y. Shatz C.. (2008). PirB is a functional receptor for myelin inhibitors of axonal regeneration. Science 322, 967–970. 10.1126/science.1161151 18988857

[B6] Bandtlow C. E. Dlaska M. Pirker S. Czech T. Baumgartner C. Sperk G. (2004). Increased expression of Nogo-A in hippocampal neurons of patients with temporal lobe epilepsy. Eur. J. Neurosci. 20, 195–206. 10.1111/j.1460-9568.2004.03470.x 15245492

[B7] Bertelsen B. Melchior L. Jensen L. R. Groth C. Nazaryan L. Debes N. M.. (2015). A t(3;9)(q25.1;q34.3) translocation leading to OLFM1 fusion transcripts in Gilles de la Tourette syndrome, OCD and ADHD. Psychiatry Res. 225, 268–275. 10.1016/j.psychres.2014.12.028 25595337

[B8] Bhagat S. M. Butler S. S. Taylor J. R. McEwen B. S. Strittmatter S. M. (2016). Erasure of fear memories is prevented by Nogo Receptor 1 in adulthood. Mol. Psychiatry 21, 1281–1289. 10.1038/mp.2015.179 26619810 PMC4887429

[B9] Budel S. Padukkavidana T. Liu B. P. Feng Z. Hu F. Johnson S.. (2008). Genetic variants of Nogo-66 receptor with possible association to schizophrenia block myelin inhibition of axon growth. J. Neurosci. 28, 13161–13172. 10.1523/JNEUROSCI.3828-08.2008 19052207 PMC2892845

[B10] Caroni P. Schwab M. (1988). Antibody against myelin-associated inhibitor of neurite growth neutralizes nonpermissive substrate properties of CNS white matter. Neuron 1, 85–96. 10.1016/0896-6273(88)90212-7 3272156

[B11] Chen Y. Cao B. Yang J. Wei Q. Ou R. W. Zhao B.. (2015). Analysis and meta-analysis of five polymorphisms of the LINGO1 and LINGO2 genes in Parkinson’s disease and multiple system atrophy in a Chinese population. J. Neurol. 262, 2478–2483. 10.1007/s00415-015-7870-9 26254004

[B12] Cotman C. Matthews D. Taylor D. Lynch G. (1973). Synaptic rearrangement in the dentate gyrus: histochemical evidence of adjustments after lesions in immature and adult rats. Proc. Natl. Acad. Sci. U S A 70, 3473–3477. 10.1073/pnas.70.12.3473 4519639 PMC427262

[B13] Dagerlind A. Friberg K. Bean A. Hökfelt T. (1992). Sensitive mRNA detection using unfixed tissue: combined radioactive and non-radioactive *in situ* hybridization histochemistry. Histochemistry 98, 39–49. 10.1007/bf00716936 1429016

[B14] Delekate A. Zagrebelsky M. Kramer S. Schwab M. Korte M. (2011). NogoA restricts synaptic plasticity in the adult hippocampus on a fast time scale. Proc. Natl. Acad. Sci. U S A 108, 2569–2574. 10.1073/pnas.1013322108 21262805 PMC3038770

[B15] Dickendesher T. L. Baldwin K. T. Mironova Y. A. Koriyama Y. Raiker S. J. Askew K. L.. (2012). NgR1 and NgR3 are receptors for chondroitin sulfate proteoglycans. Nat. Neurosci. 15, 703–712. 10.1038/nn.3070 22406547 PMC3337880

[B17] Endo T. Tominaga T. Olson L. (2009). Cortical changes following spinal cord injury with emphasis on the Nogo signaling system. Neuroscientist 15, 291–299. 10.1177/1073858408329508 19436077

[B18] Ernfors P. Wetmore C. Olson L. Persson H. (1990). Identification of cells in rat brain and peripheral tissues expressing mRNA for members of the nerve growth factor family. Neuron 5, 511–526. 10.1016/0896-6273(90)90090-3 2206535

[B19] Ferraro G. B. Morrison C. J. Overall C. M. Strittmatter S. M. Fournier A. E. (2011). Membrane-type matrix metalloproteinase-3 regulates neuronal responsiveness to myelin through Nogo-66 receptor 1 cleavage. J. Biol. Chem. 286, 31418–31424. 10.1074/jbc.m111.249169 21768085 PMC3173120

[B20] Fournier A. GrandPre T. Strittmatter S. (2001). Identification of a receptor mediating Nogo-66 inhibition of axonal regeneration. Nature 409, 341–346. 10.1038/35053072 11201742

[B21] Frisén J. (2016). Neurogenesis and gliogenesis in nervous system plasticity and repair. Annu. Rev. Cell Dev. Biol. 32, 127–141. 10.1146/annurev-cellbio-111315-124953 27298094

[B22] Fukata Y. Lovero K. L. Iwanaga T. Watanabe A. Yokoi N. Tabuchi K.. (2010). Disruption of LGI1-linked synaptic complex causes abnormal synaptic transmission and epilepsy. Proc. Natl. Acad. Sci. U S A 107, 3799–3804. 10.1073/pnas.0914537107 20133599 PMC2840530

[B23] Gil V. Nicolas O. Mingorance A. Ureña J. M. Tang B. L. Hirata T.. (2006). Nogo-A expression in the human hippocampus in normal aging and in Alzheimer disease. J. Neuropathol. Exp. Neurol. 65, 433–444. 10.1097/01.jnen.0000222894.59293.98 16772867

[B24] Halder R. Hennion M. Vidal R. O. Shomroni O. Rahman R. U. Rajput A.. (2016). DNA methylation changes in plasticity genes accompany the formation and maintenance of memory. Nat. Neurosci. 19, 102–110. 10.1038/nn.4194 26656643

[B25] Ineichen B. V. Plattner P. S. Good N. Martin R. Linnebank M. Schwab M. E. (2017). Nogo-A antibodies for progressive multiple sclerosis. CNS Drugs 31, 187–198. 10.1007/978-1-4471-2395-8_10 28105588

[B26] Iobbi C. Korte M. Zagrebelsky M. (2016). Nogo-66 restricts synaptic strengthening via Lingo1 and the ROCK2-cofilin pathway to control actin dynamics. Cereb. Cortex [Epub ahead of print]. 10.1093/cercor/bhw122 27166169

[B27] Jonsson G. (1980). Chemical neurotoxins as denervation tools in neurobiology. Annu. Rev. Neurosci. 3, 169–187. 10.1146/annurev.ne.03.030180.001125 6106449

[B28] Josephson A. Trifunovski A. Schéele C. Widenfalk J. Wahlestedt C. Brené S.. (2003). Activity-induced and developmental downregulation of the Nogo receptor. Cell Tissue Res. 311, 333–342. 10.1007/s00441-002-0695-8 12658441

[B29] Josephson A. Trifunovski A. Widmer H. Widenfalk J. Olson L. Spenger C. (2002). Nogo-receptor gene activity: cellular localization and developmental regulation of mRNA in mice and humans. J. Comp. Neurol. 453, 292–304. 10.1002/cne.10408 12378589

[B30] Josephson A. Widenfalk J. Widmer H. Olson L. Spenger C. (2001). NOGO mRNA expression in adult and fetal human and rat nervous tissue and in weight drop injury. Exp. Neurol. 169, 319–328. 10.1006/exnr.2001.7659 11358445

[B31] Karlén A. Karlsson T. Mattsson A. Lundströmer K. Codeluppi S. Pham T.. (2009). Nogo receptor 1 regulates formation of lasting memories. Proc. Natl. Acad. Sci. U S A 106, 20476–20481. 10.1073/pnas.0905390106 19915139 PMC2777184

[B32] Karlsson T. Koczy J. Brené S. Olson L. Josephson A. (2013). Differential conserted activity induced regulation of nogo receptors (1–3), LOTUS and Nogo mRNA in mouse brain. PLoS One 8:e60892. 10.1371/journal.pone.0060892 23593344 PMC3623931

[B33] Karlsson T. E. Smedfors G. Brodin A. T. Åberg E. Mattsson A. Högbeck I.. (2016). NgR1: a tunable sensor regulating memory formation, synaptic and dendritic plasticity. Cereb. Cortex 26, 1804–1817. 10.1093/cercor/bhw007 26838771 PMC4785958

[B34] Kellner Y. Fricke S. Kramer S. Iobbi C. Wierenga C. J. Schwab M. E.. (2016). Nogo-A controls structural plasticity at dendritic spines by rapidly modulating actin dynamics. Hippocampus 26, 816–831. 10.1002/hipo.22565 26748478

[B35] Lee H. Raiker S. J. Venkatesh K. Geary R. Robak L. R. Zhang Y.. (2008). Synaptic function for the Nogo-66 receptor NgR1: regulation of dendritic spine morphology and activity-dependent synaptic strength. J. Neurosci. 28, 2753–2765. 10.1523/JNEUROSCI.5586-07.2008 18337405 PMC6670664

[B36] Liu B. P. Fournier A. GrandPré T. Strittmatter S. M. (2002). Myelin-associated glycoprotein as a functional ligand for the Nogo-66 receptor. Science 297, 1190–1193. 10.1126/science.1073031 12089450

[B37] Lossos A. Elazar N. Lerer I. Schueler-Furman O. Fellig Y. Glick B.. (2015). Myelin-associated glycoprotein gene mutation causes Pelizaeus-Merzbacher disease-like disorder. Brain 138, 2521–2536. 10.1093/brain/awv204 26179919 PMC4643626

[B770] Lovero K. L. Fukata Y. Granger A. J. Fukata M. Nicoll R. A. (2015). The LGI1-ADAM22 protein complex directs synapse maturation through regulation of PSD-95 function. Proc. Natl. Acad. Sci. U S A 112, E4129–E4137. 10.1073/pnas.1511910112 26178195 PMC4522810

[B38] Lu B. Nagappan G. Lu Y. (2014). BDNF and synaptic plasticity, cognitive function and dysfunction. Handb. Exp. Pharmacol. 220, 223–250. 10.1007/978-3-642-45106-5_9 24668475

[B39] Mathews D. H. Burkard M. E. Freier S. M. Wyatt J. R. Turner D. H. (1999). Predicting oligonucleotide affinity to nucleic acid targets. RNA 5, 1458–1469. 10.1017/s1355838299991148 10580474 PMC1369867

[B40] McGee A. W. Yang Y. Fischer Q. S. Daw N. W. Strittmatter S. M. (2005). Experience-driven plasticity of visual cortex limited by myelin and Nogo receptor. Science 309, 2222–2226. 10.1126/science.1114362 16195464 PMC2856689

[B41] Meabon J. S. De Laat R. Ieguchi K. Wiley J. C. Hudson M. P. Bothwell M. (2015). LINGO-1 protein interacts with the p75 neurotrophin receptor in intracellular membrane compartments. J. Biol. Chem. 290, 9511–9520. 10.1074/jbc.M114.608018 25666623 PMC4392256

[B42] Meininger V. Pradat P. F. Corse A. Al-Sarraj S. Rix Brooks B. Caress J. B.. (2014). Safety, pharmacokinetic and functional effects of the nogo-a monoclonal antibody in amyotrophic lateral sclerosis: a randomized, first-in-human clinical trial. PLoS One 9:e97803. 10.1371/journal.pone.0097803 24841795 PMC4026380

[B43] Mi S. Lee X. Shao Z. Thill G. Ji B. Relton J.. (2004). LINGO-1 is a component of the Nogo-66 receptor/p75 signaling complex. Nat. Neurosci. 7, 221–228. 10.1038/nn1188 14966521

[B44] Mironova Y. A. Giger R. J. (2013). Where no synapses go: gatekeepers of circuit remodeling and synaptic strength. Trends Neurosci. 36, 363–373. 10.1016/j.tins.2013.04.003 23642707 PMC3674226

[B45] Muona M. Fukata Y. Anttonen A. K. Laari A. Palotie A. Pihko H.. (2016). Dysfunctional ADAM22 implicated in progressive encephalopathy with cortical atrophy and epilepsy. Neurol. Genet. 2:e46. 10.1212/NXG.0000000000000046 27066583 PMC4817901

[B46] Nakaya N. Sultana A. Lee H. -S., Tomarev S. I., (2012). Olfactomedin 1 interacts with the Nogo A receptor complex to regulate axon growth. J. Biol. Chem. 287, 37171–37184. 10.1074/jbc.M112.389916 22923615 PMC3481317

[B47] Nordgren M. Karlsson T. Svensson M. Koczy J. Josephson A. Olson L.. (2013). Orchestrated regulation of Nogo receptors, LOTUS, AMPA receptors and BDNF in an ECT model suggests opening and closure of a window of synaptic plasticity. PLoS One 8:e78778. 10.1371/journal.pone.0078778 24244357 PMC3828303

[B48] Novak G. Kim D. Seeman P. Tallerico T. (2002). Schizophrenia and Nogo: elevated mRNA in cortex and high prevalence of a homozygous CAA insert. Brain Res. Mol. 107, 183–189. 10.1016/s0169-328x(02)00492-8 12425946

[B49] Park J. Gimbel D. GrandPre T. Lee J. Kim J. Li W.. (2006a). Alzheimer precursor protein interaction with the Nogo-66 receptor reduces amyloid-*β* plaque deposition. J. Neurosci. 26, 1386–1395. 10.1523/JNEUROSCI.3291-05.2006 16452662 PMC2846286

[B50] Park J. Widi G. Gimbel D. Harel N. Lee D. Strittmatter S. (2006b). Subcutaneous Nogo receptor removes brain amyloid-β and improves spatial memory in Alzheimer’s transgenic mice. J. Neurosci. 26, 13279–13286. 10.1523/JNEUROSCI.4504-06.2006 17182778 PMC2856604

[B51] Park J. Yiu G. Kaneko S. Wang J. Chang J. He X.. (2005). A TNF receptor family member, TROY, is a coreceptor with Nogo receptor in mediating the inhibitory activity of myelin inhibitors. Neuron 45, 345–351. 10.1016/j.neuron.2004.12.040 15694321

[B52] Péan S. (2012). Available online at: http://www.samuelpean.com/heatmap-histogram/

[B53] Petrasek T. Prokopova I. Bahnik S. Schonig K. Berger S. Vales K.. (2014a). Nogo-A downregulation impairs place avoidance in the Carousel maze but not spatial memory in the Morris water maze. Neurobiol. Learn. Mem. 107, 42–49. 10.1016/j.nlm.2013.10.015 24211256

[B54] Petrasek T. Prokopova I. Sladek M. Weissova K. Vojtechova I. Bahnik S.. (2014b). Nogo-A-deficient transgenic rats show deficits in higher cognitive functions, decreased anxiety and altered circadian activity patterns. Front. Behav. Neurosci. 8:90. 10.3389/fnbeh.2014.00442 24672453 PMC3957197

[B55] Poo M. M. Pignatelli M. Ryan T. J. Tonegawa S. Bonhoeffer T. Martin K. C.. (2016). What is memory? The present state of the engram. BMC Biol. 14:40. 10.1186/s12915-016-0261-6 27197636 PMC4874022

[B56] Raisman G. (1969). Neuronal plasticity in the septal nuclei of the adult rat. Brain Res. 14, 25–48. 10.1016/0006-8993(69)90029-8 5783115

[B57] Robak L. A. Venkatesh K. Lee H. Raiker S. Duan Y. Lee-Osbourne J.. (2009). Molecular basis of the interactions of the Nogo-66 receptor and its homolog NgR2 with myelin-associated glycoprotein: development of NgROMNI-Fc, a novel antagonist of CNS myelin inhibition. J. Neurosci. 29, 5768–5783. 10.1523/JNEUROSCI.4935-08.2009 19420245 PMC2779053

[B58] Sato Y. Iketani M. Kurihara Y. Yamaguchi M. Yamashita N. Nakamura F.. (2011). Cartilage acidic protein-1B (LOTUS), an endogenous Nogo receptor antagonist for axon tract formation. Science 333, 769–773. 10.1126/science.1204144 21817055 PMC3244695

[B59] Satoh J. Onoue H. Arima K. Yamamura T. (2005). Nogo-A and nogo receptor expression in demyelinating lesions of multiple sclerosis. J. Neuropathol. Exp. Neurol. 64, 129–138. 10.1093/jnen/64.2.129 15751227

[B60] Schauwecker P. E. Steward O. (1997). Genetic determinants of susceptibility to excitotoxic cell death: implications for gene targeting approaches. Proc. Natl. Acad. Sci. U S A 94, 4103–4108. 10.1073/pnas.94.8.4103 9108112 PMC20575

[B61] Schwab M. E. (2010). Functions of Nogo proteins and their receptors in the nervous system. Nat. Rev. Neurosci. 11, 799–811. 10.1038/nrn2936 21045861

[B771] Senechal K. R. Thaller C. Noebels J. L. (2005). ADPEAF mutations reduce levels of secreted LGI1, a putative tumor suppressor protein linked to epilepsy. Hum. Mol. Genet. 14, 1613–1620. 10.1093/hmg/ddi169 15857855

[B62] Snyder D. A. Rivers A. M. Yokoe H. Menco B. P. Anholt R. R. (1991). Olfactomedin: purification, characterization and localization of a novel olfactory glycoprotein. Biochemistry 30, 9143–9153. 10.1021/bi00102a004 1892825

[B64] Stephany C. É. Frantz M. G. McGee A. W. (2016a). Multiple roles for nogo receptor 1 in visual system plasticity. Neuroscientist 22, 653–666. 10.1177/1073858415614564 26552866 PMC6592612

[B65] Stephany C. É. Ikrar T. Nguyen C. Xu X. McGee A. W. (2016b). Nogo receptor 1 confines a disinhibitory microcircuit to the critical period in visual cortex. J. Neurosci. 36, 11006–11012. 10.1523/JNEUROSCI.0935-16.2016 27798181 PMC5098837

[B66] Tews B. Schonig K. Arzt M. E. Clementi S. Rioult-Pedotti M. S. Zemmar A.. (2013). Synthetic microRNA-mediated downregulation of Nogo-A in transgenic rats reveals its role as regulator of synaptic plasticity and cognitive function. Proc. Natl. Acad. Sci. U S A 110, 6583–6588. 10.1073/pnas.1217665110 23576723 PMC3631667

[B68] Thomas R. A. Ambalavanan A. Rouleau G. A. Barker P. A. (2016). Identification of genetic variants of LGI1 and RTN4R (NgR1) linked to schizophrenia that are defective in NgR1-LGI1 signaling. Mol. Genet. Genomic Med. 4, 447–456. 10.1002/mgg3.215 27468420 PMC4947863

[B67] Thomas R. A. Favell K. Morante-Redolat J. Pool M. Kent C. Wright M.. (2010). LGI1 is a Nogo receptor 1 ligand that antagonizes myelin-based growth inhibition. J. Neurosci. 30, 6607–6612. 10.1523/JNEUROSCI.5147-09.2010 20463223 PMC6632578

[B69] Trifunovski A. Josephson A. Ringman A. Brené S. Spenger C. Olson L. (2004). Neuronal activity-induced regulation of Lingo-1. Neuroreport 15, 2397–2400. 10.1097/00001756-200410250-00019 15640763

[B70] Venkatesh K. Chivatakarn O. Lee H. Joshi P. Kantor D. Newman B.. (2005). The Nogo-66 receptor homolog NgR2 is a sialic acid-dependent receptor selective for myelin-associated glycoprotein. J. Neurosci. 25, 808–822. 10.1523/JNEUROSCI.4464-04.2005 15673660 PMC6725623

[B71] Wang K. C. Koprivica V. Kim J. A. Sivasankaran R. Guo Y. Neve R. L.. (2002). Oligodendrocyte-myelin glycoprotein is a Nogo receptor ligand that inhibits neurite outgrowth. Nature 417, 941–944. 10.1038/nature00867 12068310

[B72] Willi R. Schwab M. E. (2013). Nogo and Nogo receptor: relevance to schizophrenia? Neurobiol. Dis. 54, 150–157. 10.1016/j.nbd.2013.01.011 23369871

[B73] Wills Z. Mandel-Brehm C. Mardinly A. McCord A. Giger R. Greenberg M. (2012). The nogo receptor family restricts synapse number in the developing hippocampus. Neuron 73, 466–481. 10.1016/j.neuron.2011.11.029 22325200 PMC3532882

[B74] Yang G. Pan F. Gan W. (2009). Stably maintained dendritic spines are associated with lifelong memories. Nature 462, 920–924. 10.1038/nature08577 19946265 PMC4724802

[B75] Yuste R. Bonhoeffer T. (2001). Morphological changes in dendritic spines associated with long-term synaptic plasticity. Annu. Rev. Neurosci. 24, 1071–1089. 10.1146/annurev.neuro.24.1.1071 11520928

[B76] Zafra F. Lindholm D. Castren E. Hartikka J. Thoenen H. (1992). Regulation of brain-derived neurotrophic factor and nerve growth factor mRNA in primary cultures of hippocampal neurons and astrocytes. J. Neurosci. 12, 4793–4799. 1281495 10.1523/JNEUROSCI.12-12-04793.1992 PMC6575775

[B77] Zagrebelsky M. Lonnemann N. Fricke S. Kellner Y. Preuß E. Michaelsen-Preusse K.. (2017). Nogo-A regulates spatial learning as well as memory formation and modulates structural plasticity in the adult mouse hippocampus. Neurobiol. Learn. Mem. 138, 154–163. 10.1016/j.nlm.2016.06.022 27349794

[B78] Zemmar A. Weinmann O. Kellner Y. Yu X. Vicente R. Gullo M.. (2014). Neutralization of nogo-a enhances synaptic plasticity in the rodent motor cortex and improves motor learning *in vivo*. J. Neurosci. 34, 8685–8698. 10.1523/JNEUROSCI.3817-13.2014 24966370 PMC4147625

[B79] Zuker M. (2003). Mfold web server for nucleic acid folding and hybridization prediction. Nucleic Acids Res. 31, 3406–3415. 10.1093/nar/gkg595 12824337 PMC169194

